# Clarifying Vaccination Status, Response Rate, and Regression Estimates in a National COVID‐19 Survey From the Democratic Republic of the Congo

**DOI:** 10.1002/hsr2.73141

**Published:** 2026-09-02

**Authors:** Muhammad Mujtaba Rasool, Muhammad Murtaza, Areesha Imtiaz, Arwa Adnan, Hamza Khurram, Ali Jan Yaqubi

**Affiliations:** ^1^ Department of Medicine Allama Iqbal Medical College Lahore Pakistan; ^2^ Department of Medicine Services Institute of Medical Sciences Lahore Pakistan; ^3^ Department of Medicine Jinnah Sindh Medical University Karachi Pakistan; ^4^ Department of Medicine Karachi Medical and Dental College Karachi Pakistan; ^5^ Department of Medicine Kabul University of Medical Sciences kabul Afghanistan

Dear Editor,

We read with interest the national telephone survey by Bosonkie et al. examining COVID‐19 vaccine uptake and intention to vaccinate among adults in the Democratic Republic of the Congo (DRC) [1]. The multilingual survey, quota‐based recruitment across all 26 provinces, interviewer training, back‐translation, and use of modified Poisson regression are notable strengths. Several inconsistencies in outcome classification and numerical reporting, however, require clarification because they affect the interpretation and reproducibility of the principal estimates.

The most important issue concerns the definition of vaccination status. The Methods state that participants receiving fewer than two doses of an mRNA vaccine were classified as having an incomplete dose and, “in the last case, they were not vaccinated.” [1] Table 2 nevertheless treats incomplete vaccination as a separate category: 89 participants were fully vaccinated, 38 incompletely vaccinated, and 948 not vaccinated. More importantly, the “Already took COVID‐19 vaccine” outcome in Table 3 appears to include participants with any dose rather than only those meeting the stated full‐vaccination definition. Across sex categories, 126 participants are classified as vaccinated, closely approximating the 127 participants with either a full or incomplete dose (89 + 38), with one participant apparently missing sex data. Conversely, the intention analysis is restricted to the 948 participants labeled “not vaccinated,” excluding the 38 incomplete‐dose recipients despite the Methods stating that these participants were not vaccinated. These classifications answer different epidemiological questions and can produce different prevalence ratios. The vaccine‐product definition also requires clarification: the Introduction notes that AstraZeneca was introduced through COVAX in the DRC, yet the vaccination definition specifies only Pfizer, Moderna, and Johnson & Johnson. WHO guidance in effect during the survey described AstraZeneca as a two‐dose primary series [2]. The authors should therefore specify how AstraZeneca recipients and incomplete‐dose recipients were coded in each analysis.

Several regression‐table values are also internally incompatible. In Table 4, the adjusted prevalence ratio for vaccination intention among participants aged 36–55 years is reported as 1.21 with a 95% confidence interval of 0.93–1.12; a point estimate cannot lie outside its own confidence interval [1]. For participants aged 56 years or older, the table reports 57 intending vaccination and 17 not intending vaccination, which corresponds to 77.0% (57/74), rather than the printed 70.1%. For high socioeconomic status, the displayed proportions are 65.0% versus 76.9% in the low‐status reference group, giving a crude prevalence ratio of approximately 0.85, rather than the reported 0.76. Education coding appears to shift as well: Table 1 reports 252 participants with secondary‐ordinary education and 465 with secondary‐advanced education, whereas Table 3 contains totals of 464 and 252 for the corresponding labels. In Table 4, the denominators again align with the opposite labels. Corrected tables and model outputs would allow readers to determine whether these are transcription errors or reflect differences in analytic coding.

The reported 98.3% “response rate” also warrants reconsideration. The prespecified target was 1093 participants and 1075 were enrolled; 1075/1093 equals 98.3% [1]. This is the proportion of the target sample achieved, not, by itself, a survey response rate. The sampling plan anticipated approximately 5465 working telephone numbers, yet the article does not report the number actually dialled, unanswered, ineligible, refusing, partially completing, or completing the interview. Standard survey response rates require disposition information for sampled cases [3]. Consequently, the statement that 98.3% of participants consented and that this ensured greater representativeness cannot be independently verified. This is particularly relevant because quotas were based partly on the national distribution of reported COVID‐19 cases rather than the adult population distribution, and no population weighting procedure is described.

Relatedly, model‐specific denominators and missing‐data handling are unclear. Table 1 includes “not stated” categories for education, religion, and occupation, while the totals entering Tables 3 and 4 vary by covariate without an explicit description of complete‐case exclusions or other missing‐data handling [1]. STROBE recommends reporting the number of participants with missing data for each variable of interest and explaining how missing data were addressed [4]. Providing analytic sample sizes for each model would help distinguish true missingness from coding or table‐assembly errors.

Finally, the abstract should be harmonized with the main results. The health‐worker estimate is reported as adjusted PR 1.62 with “95% CI: 2.34,” omitting the lower confidence limit, while Table 3 reports 1.12–2.34. The age estimate is reported as 1.05–3.27 in the abstract but 1.07–3.27 in the Results and Table 3 [1]. In the Discussion, the statement that “8 out of 10” vaccinated participants were vaccinated to protect themselves also conflicts with Figure 1, which reports 60.8%. These may be editorial errors, but correction is important because the abstract and discussion are often the most widely read sections.

Bosonkie et al. provide valuable national data from a difficult survey setting. Clarifying the vaccination outcome, correcting the regression tables, reporting true call dispositions and model‐specific denominators, and harmonizing the abstract with the main analysis would materially strengthen the transparency and interpretability of the study.

## Author Contributions

**Muhammad Mujtaba Rasool:** conceptualization, methodology, literature review, writing – original draft, and project administration. **Muhammad Murtaza:** conceptualization, supervision, validation, and writing – review and editing. **Areesha Imtiaz:** literature review, data verification, and writing – review and editing. **Arwa Adnan:** literature review, methodological appraisal, and reference validation. **Hamza Khurram:** statistical appraisal, data verification, and critical revision of the manuscript. **Ali Jan Yaqubi:** validation, critical revision, and writing – review and editing. All authors reviewed and approved the final version of the manuscript and accept responsibility for its content.

## Funding

The authors have nothing to report.

## Conflicts of Interest

The authors declare no conflicts of interest.

## Data Availability

Data sharing not applicable to this article as no datasets were generated or analyzed during the current study. Data sharing is not applicable to this article because no new data were generated or analyzed.
